# A controlled trial of the effectiveness of internet continuing medical education

**DOI:** 10.1186/1741-7015-6-37

**Published:** 2008-12-04

**Authors:** Linda Casebeer, Sally Engler, Nancy Bennett, Martin Irvine, Destry Sulkes, Marc DesLauriers, Sijian Zhang

**Affiliations:** 1 Outcomes, Inc., 300 Riverchase Pkwy E, Birmingham, AL 35244, USA; 2 Consultant, Outcomes, Inc., 300 Riverchase Pkwy E, Birmingham, AL 35244, USA; 3 Medscape, LLC, 76 Ninth Avenue, New York, NY 10011, USA; 4 University of Alabama at Birmingham, Birmingham, AL, USA

## Abstract

**Background:**

The internet has had a strong impact on how physicians access information and on the development of continuing medical education activities. Evaluation of the effectiveness of these activities has lagged behind their development.

**Methods:**

To determine the effectiveness of a group of 48 internet continuing medical education (CME) activities, case vignette surveys were administered to US physicians immediately following participation, and to a representative control group of non-participant physicians. Responses to case vignettes were analyzed based on evidence presented in the content of CME activities. An effect size for each activity was calculated using Cohen's *d *to determine the amount of difference between the two groups in the likelihood of making evidence-based clinical decisions, expressed as the percentage of non-overlap, between the two groups. Two formats were compared.

**Results:**

In a sample of 5621 US physicians, of the more than 100,000 physicians who participated in 48 internet CME activities, the average effect size was 0.75, an increased likelihood of 45% that participants were making choices in response to clinical case vignettes based on clinical evidence. This likelihood was higher in interactive case-based activities, 51% (effect size 0.89), than for text-based clinical updates, 40% (effect size 0.63). Effectiveness was also higher among primary care physicians than specialists.

**Conclusion:**

Physicians who participated in selected internet CME activities were more likely to make evidence-based clinical choices than non-participants in response to clinical case vignettes. Internet CME activities show promise in offering a searchable, credible, available on-demand, high-impact source of CME for physicians.

## Background

The internet has had a strong impact on how physicians access information, and many have reported the influence of this information on their medical decision making [1,2]. The internet offers a platform for addressing healthcare quality and patient safety by assisting with diagnosis and patient management, and facilitating the free flow of information [3]. The internet also offers opportunities to facilitate improvement in the quality of care through physician maintenance of certification [4,5].

Rapid growth of the internet has altered continuing education for health professionals by allowing access to more varied, individualized, and systematic educational opportunities. In 2008, 300 sites offered more than 16,000 CME activities [6]. Internet CME activities offer advantages over traditional methods of CME delivery; internet CME is a credible 'any time, any place' form of education, providing increased accessibility to busy physicians [7-11]. Other advantages may include increased engagement in the educational process, ease of use, cost effectiveness, hyperlinked navigation, and the ability to view content that may be continually updated.

The evaluation of internet CME activities has not kept pace with their development; evaluation has principally focused on participant satisfaction and increases in knowledge [12,13]. Only a few studies have examined physician performance and patient health associated with participation in internet CME activities, and the results have been mixed [14-18]. Evaluation studies of internet CME activities have been limited by the lack of systematic evaluation across different clinical subject matter areas [12]. The purpose of this study was to use a consistent approach to evaluate the effectiveness of internet CME activities across various clinical topics by examining the amount of difference in the evidence-based clinical practice choices of participants compared with a control group of non-participants. Based on a recent meta-analysis of the effectiveness of CME activities [19], we hypothesized that physicians participating in internet CME activities would make evidence-based clinical practice choices more frequently than physicians who did not participate, and that the percentage of non-overlap in evidence-based choices between the two groups would be at least 10%.

## Methods

A controlled trial was designed to measure the effectiveness of a group of 48 internet CME activities. Physicians who participated in these activities, matched the target audience for the activity, and completed case vignette self-assessment questions following participation were eligible to participate. A random sample of participants meeting the eligible criteria for each activity was drawn from each overall group. A random sample of non-participant physicians of similar specialties was identified as a control group and was asked to complete the same self-assessment questions. The average evidence-based response rates were calculated for the participant and non-participant samples for each activity, and an effect size was calculated. An overall effect size was calculated, as well as effect sizes for text and case-based activities, and for primary care and specialist participants.

A consistent assessment approach was developed that included 1) using case vignettes to assess clinical practice choices, 2) using a standard hypertext mark-up language programming approach to presenting assessment questions at the end of selected internet activities, 3) applying this assessment approach to specific content reflected in each individual activity, 4) collecting assessment data from CME participants in each individual clinical assessment, 5) collecting assessment data from a comparable group of non-participants in each of the assessments, and 6) analyzing the data to determine the amount of difference between the CME participant and non-participant groups by calculating effect size and the percentage of non-overlap between the two groups. The use of case vignette surveys was reviewed by the Western Institutional Review Board in 2004, prior to initiation of this study; voluntary completion of the survey questions by physicians was considered to constitute consent.

During 2005, a pilot was conducted on three internet CME activities to test a standardized evaluation procedure, and the use of standard hypertext mark-up language (HTML) online forms, for the purpose of systematically gathering clinical case vignette assessment data from physicians following participation in internet CME activities posted on a large medical education site. The pilot was designed to determine the technical feasibility of gathering and transferring large data sets using a standardized evaluation approach; the pilot was not designed to evaluate the effectiveness of the three internet CME activities. The standardized evaluation procedure included the following elements. A standard assessment template consisting of two clinical vignettes and five clinical questions was developed using a multiple choice format; evidence-based responses to the case vignettes were identified from content and references developed by the faculty for each activity. Content for the activities was written and referenced to clinical evidence by the faculty member for each activity. Only content referenced to peer-reviewed publications or guidelines was considered eligible for the development of clinical vignette assessment questions. Case vignettes were written by physicians and were referenced to the content and learning objectives. Content validity of the case vignettes was established by review from medical editors of the online portal; editors represented the appropriate clinical area for each set of case vignettes.

Case vignette evaluations were developed for the three pilot activities according to this procedure. Over 5000 physicians participated in the pilot activities. Data collection and transfer was successful; no technical glitches were identified in data collection using the HTML online forms or in the data transfer. This feasibility pilot established the processes for development and review of case vignette questions, as well as the technical platform for proceeding with the evaluation of the effectiveness of a series of 48 internet CME activities.

During an 18-month period, a group of internet CME activities was identified as eligible for assessment if the activity met the following criteria: 1) designed for physicians, 2) posted during an 18 month period between January 2006 and June 2007 to a large medical education website, 3) certified for CME credit, 4) presented in an on-demand archived format (webcasts and other live activities were not included), and 5) designed in a text-based format for clinical updates or as interactive case-based activities.

Text-based clinical update activities were defined as original review articles on scientific advances related to a particular clinical topic, similar to a written article in an internet journal. Interactive cases were original CME activities presented in a case format with extensive questions and feedback within each activity. Typically, they began with a short explanatory introduction and then presented the content within the context of a patient care scenario with discussion of diagnostic and therapeutic options and outcomes. Questions distributed throughout the activity provided interaction for learners to test their knowledge on either the material that was just presented, or for upcoming content. After submitting a response, the learner was presented with an explanation of the optimal answer, as well as a summary of the responses of past participants. There was no direct learner-instructor or learner-learner interaction in either of these formats.

The case vignette survey template consisted of a set of content-specific, case vignette questions that were delivered to participants at the conclusion of each CME activity. They were also distributed in a survey, by email or fax, to a similar non-participant group. This method was chosen as an adaptation for an online format with automated data transfer of the case vignette assessment method that has been recognized for its value in predicting physician practice patterns; results from recent research demonstrate case vignettes, compared with other processes of care measures such as chart review and standardized patients, are a valid and comprehensive method to measure a physician's processes of care [20,21].

A sample size of at least 4800 with at least 100 (50 participants and 50 non-participants selected as a desired minimum sample size for individual activities) for each of the CME activities was chosen for the study in order to establish consistency in data collection even though content varied across multiple clinical areas. Participants were eligible for inclusion in the study only if they represented the specialty target audience for the activity, or were providing primary care. Eligible participants were identified for each activity, and a random sample of 50 was drawn from the group of eligible participants. Non-participating physicians were identified from a random sample drawn by specialty from the physician list of the American Medical Association. Participant and non-participant samples were matched on the following characteristics: physician specialty, degree, years in practice, whether or not direct patient care was their primary responsibility, and the average number of patients seen per week.

A statistical analysis software package (SAS 9.1.3) was used in data extraction and transformation, and statistical analyses. Participant and non-participant case vignette responses were scored according to their concordance with the evidence-informed content presented within each activity. Overall mean scores and pooled standard deviations were calculated for both the participant and non-participant groups for each of the activities. These were used to calculate the educational effect size using Cohen's *d *formula (i.e., the difference in mean divided by the square root of the pooled standard deviation) in order to determine the average amount of difference between participants and non-participants [22]. Effect size representing the difference between the two groups was expressed as a percentage of non-overlap between participants and non-participants. The amount of difference between participants and non-participants in the likelihood of making evidence-based clinical choices in response to clinical case vignettes was expressed using the percentage of non-overlap between participants and non-participants for each activity, and for the overall group of activities.

## Results

Over 100,000 US physicians participated in the 48 selected activities over an 18 month period. A total of 5621 physician responses to assessment questions in 48 activities were analyzed; of these, 2785 physicians were responses from CME participants and 2836 were received from the control group of non-participants. The CME participant sample represents 1377 primary care physicians and 1241 physicians specializing in other areas. The non-participant sample represents 1441 primary care physicians and 1270 physicians specializing in other areas of medicine.

Demographics of physicians specializing in primary care, of physicians specializing in other clinical areas, and of all respondents, are presented in Table 1. Demographics of the participant group were consistent with demographics of the US physician population except in regard to patient care as a principal responsibility. Nationally, the average age of physicians is 51 years, with 27.8% female physicians and 6.5% representing those with DO degrees [23]. Nationally, 78.4% of US physicians are primarily involved with patient care; in the participant sample, this was significantly higher, at 94% [23]. When primary care participants were compared with specialist participants, there were no significant differences except in regard to gender. Primary care physician participants were more likely to be female (33%), compared with specialist participants (21%).

**Table 1:** Demographics of physician internet CME participants and non-participants

**Characteristics** **All physicians **(*N *= 5621)	**Participant** (*N *= 2785)	**Non-participant** (*N *= 2836)
Age, years		
Average	53.0	51.3
SD	10.0	8.1
Years since graduation		
Average	26.1	24.6
SD	10.3	8.4
Gender, number (%)		
Female	736 (27)	537 (21)
Male	2003 (73)	1964 (79)
Degree, number (%)		
DO	116 (4)	130 (5)
MD	2668 (96)	2688 (95)
Direct patient care as principal responsibility, number (%)	2572 (94)	2621 (94)

Of the 48 internet CME activities posted during the 18 month period of the study, 24 were interactive CME cases and 24 were text-based clinical updates. Effect sizes were highest for the cardiology and neurology activities. The effect sizes for these activities are presented in Tables 2 and 3 by clinical area and activity type.

**Table 2:** Interactive CME cases

**CME ACTIVITY**	**Participants**			**Non-participants**			**Effect size**
	***N***	**Mean % evidence-based responses**	**SD**	***N***	**Mean % evidence-based responses**	**SD**	
**Psychiatry**							
The diagnosis and treatment of schizophrenia	33	66	15	46	53	20	0.75
Generalized Anxiety Disorder	32	66	21	26	56	15	0.56
Attention Deficit Hyperactivity Disorder case studies	32	91	13	33	82	18	0.60
The evolving face of Attention Deficit Hyperactivity Disorder	49	78	23	45	54	25	0.59
Diagnosis/Management of Attention Deficit Hyperactivity Disorder	36	78	19	36	60	18	0.98
**Neurology**							
Advances in Restless Leg Syndrome	64	89	16	58	55	24	1.68
Advances in stroke	77	91	11	58	63	19	1.89
Emerging concepts in the treatment of multiple sclerosis	21	62	19	25	46	17	0.89
**Cardiology**							
Pulmonary arterial hypertension	32	84	14	31	58	18	1.61
Predicting heart failure	26	71	24	28	49	19	1.02
Hypertension highlights	101	73	27	92	49	25	0.91
**Rheumatology**							
Managing the rheumatoid arthritis patient	48	81	18	49	68	25	0.60
Early and aggressive treatment of rheumatoid arthritis	38	71	22	39	43	27	1.15
Assessment of the patient with rheumatoid arthritis	17	74	23	17	47	16	1.37
Safety of biologic agents in the treatment of rheumatoid arthritis	22	86	15	18	59	23	1.38
**Urology**							
Urinary frequency in an elderly woman	60	93	13	52	78	19	0.95
Improving outcomes in benign prostatic hyperplasia	68	78	20	50	62	15	0.90
Obesity, erectile dysfunction and hypertension	60	85	18	60	48	30	1.49
Treating overactive bladder in the elderly patient	103	76	22	95	55	27	0.84
**Infectious disease**							
Antimicrobial resistance	49	70	23	49	42	19	1.34
Emergent presentation and treatment of influenza in children	69	76	20	65	61	22	0.72
**Pulmonology**							
Diagnosis/treatment of asthma in infants and young children	60	90	13	61	60	20	1.70
Diagnosis of Chronic Obstructive Pulmonary Disorder and asthma	17	92	16	17	74	15	1.13
**Women's health**							
Contraception in a woman with a history of fibroids	53	79	22	47	68	21	0.52

**Table 3:** Effect size of text-based clinical updates

**CME ACTIVITY**	**Participants**			**Non-participants**			**Effect size**
	***N***	**Mean % evidence-based response**	**SD**	***N***	**Mean % evidence-based response**	**SD**	
**Psychiatry**							
Identifying patients with prodromal schizophrenia	35	73	12	28	49	16	1.76
Treatment nonadherence among individuals with schizophrenia	52	71	17	47	54	21	0.90
Treatment resistant schizophrenia	50	77	18	50	60	15	1.08
Reducing suicide risk in patients with schizophrenia	49	81	21	46	59	27	0.92
Recognizing and preventing abuse of rapid-onset opioids	50	68	21	57	48	21	0.93
Detecting autism in a toddler	100	68	19	102	50	22	0.85
Hyperactivity or autism	101	61	19	95	47	20	0.73
**Infectious disease**							
Maximizing therapeutic success in an era of increasing antibiotic resistance	43	73	27	43	51	28	0.80
The role of antibiotics in serious hospital acquired infections	47	87	20	54	71	19	0.82
Fungal infection in the immunocompromised patient	47	68	21	39	54	21	0.65
**Urology**							
Prostate cancer	26	71	24	28	49	19	0.28
Lower urinary tract symptoms in men	24	74	23	24	58	20	0.77
Evaluation and treatment of overactive bladder	41	74	29	45	63	28	0.37
Erectile dysfunction and cardiovascular disease	29	63	14	25	57	22	0.32
**Cardiology**							
Statin efficacy and prevention of recurrent stroke	22	83	11	22	60	16	1.62
Diabetes, Metabolic Syndrome and very low cholesterol	59	88	21	59	72	30	0.62
Statins in the treatment of heart failure	50	60	18	49	45	19	0.83
Advances in hypertension	22	82	18	22	58	27	1.04
Medical adherence	22	55	22	22	38	16	0.89
Analysis of latest trial data points to new guidelines & restructured hypertension therapy	50	85	19	49	59	25	1.04
**Neurology**							
The role of MRI in multiple sclerosis diagnosis and management	53	64	21	53	46	19	0.89
Breakthrough pain	73	75	27	73	48	29	0.98
**Rheumatology**							
Risks and benefits of COX-2 selective inhibitors	85	70	23	98	62	24	0.36
**Women's health**							
Contraception today	18	79	21	21	57	20	1.12

Overall, the average effect size for the 48 internet CME activities was 0.75. (Table 4) The non-overlap percentile, representing the non-overlap between participants and non-participants in evidence-based responses, was 45.2%, exceeding the hypothesized non-overlap of 10% between the two groups. Interactive case-based internet CME activities demonstrated a significantly higher effect size than text-based programming (*p *= 0.001). The effect size for primary care participants was also significantly higher than that for specialists. (*p *= < 0.001).

**Table 4:** Effect size of 48 internet CME activities by format and specialty

	***N***	**Effect size**	**% of non-overlap between participants and non-participants**
All 48 internet CME activities	5621	0.75	45.2%
24 text-based internet CME activities	2780	0.63	39.8%
24 case-based internet CME activities	2841	0.89	51.0%
All activities/primary care participants	2818	0.83	48.5%
All activities/specialist participants	2511	0.70	43.0%

## Discussion

Physician participants using internet CME activities selected evidence-based choices for clinical care in response to case vignettes more frequently than non-participants. The likelihood that physician internet CME participants would make clinical choices consistent with evidence in response to case vignettes more often than non-participants greatly exceeded the hypothesized 10% non-overlap between the two groups, demonstrating instead a 45% non-overlap. This effect is stronger than that from a recent meta-analysis of the effectiveness of CME activities where CME interventions had a small to moderate effect on physician knowledge and performance [19]. In this meta-analysis, however, only two internet studies were included.

The somewhat higher effect size for primary care physicians may be a reflection of broader educational needs, due to the wide range of clinical problems they encounter. Physicians specializing in clinical areas other than primary care have a narrower focus for medical information seeking and may have higher levels of baseline knowledge than primary care physicians on specific topics, also contributing to differences in effect size. The higher effect size for interactive CME cases is consistent with previous studies that demonstrate that increases in active participation improve the effectiveness of CME [24].

Internet CME physician participants represented by the sample in this study have extensive experience, and they are principally engaged in direct patient care, disputing earlier perceptions that most physicians accessing internet CME would be recent graduates from medical school. Compared with demographic data on the total population of US physicians, the years in practice are similar, but more physicians in the online group are engaged principally in patient care [23]. A higher percentage of female physicians participated in the online CME activities studied than is represented in the overall US physician population. It is clear that internet CME activities are reaching a large audience of busy physicians; the ACCME data compilation for 2006 showed that physicians participated in internet enduring materials over 2 million times [25]. Data from this study have demonstrated that, in addition to large increases in reach for internet CME, these activities show promise in influencing practice. The larger effect size for these internet CME activities may be associated with the searchability of internet CME activities, as well as their availability when physicians are prompted to address a clinical question or problem. More research is needed in this area.

One of the strengths of this study was the use of a consistent evaluation format applied to a large number of internet CME activities. A limitation, however, was the programmed format that limited the number of clinical vignette questions to five in each activity; thus, not all key points in the content of each activity could be evaluated. The format also limited the type of questions to multiple-choice questions, and did not include the opportunity to ask open-ended questions. While the use of a control group allowed a comparison of participants with non-participants, another limitation was the lack of baseline data to assess the practice patterns of CME participants prior to participation. It is possible that CME internet participants access the internet more frequently than non-participants, and access not only CME activities, but various forms of internet medical information; these medical information seeking behaviors may influence the amount of difference between participants and non-participants reflected in the effect sizes reported in this study. In future studies, baseline data would be helpful in addressing this issue.

While this study has demonstrated the promise of internet CME activities in influencing the diagnostic and therapeutic choices physicians make daily, many research questions have yet to be addressed. Future research studies should continue to apply consistent evaluation approaches to internet CME. Pre-tests or baseline measurements would contribute to a more robust understanding of physician practice patterns prior to participation; it will be important, however, not to create lengthy pre-tests that become barriers to accessing internet CME activities. Future studies are needed to determine not only which internet formats are most effective, but also how educational elements such as advance organizers, behavioral objectives, interactivity, and feedback should be incorporated into the design of activities to optimize effectiveness. In addition, studies will be needed to determine how activities can be tailored to various physician specialties and populations.

## Conclusion

In summary, evaluation of internet CME activities lags far behind the development of these activities, and many research questions remain unaddressed. This study, however, has demonstrated that physicians who participated in selected internet CME activities were more likely following participation to make evidence-based clinical choices in response to case vignettes than were non-participants. Internet CME activities show promise in offering a searchable, credible, available on-demand, high-impact source of CME for physicians.

## Competing interests

Authors are employees of Outcomes, Inc., Medscape LLC, or the University of Alabama School of Public Health. They have no other potential conflicts of interest to declare.

## Authors' contributions

All authors have participated in the design of the study, the review of the data, and the writing of the article. SZ was principally responsible for the data analysis.

## Pre-publication history

The pre-publication history for this paper can be accessed here:


